# Oxidative Stress and Keap1-Nrf2 Pathway Involvement in Bisphenol A-Induced Liver Damage in Rats

**DOI:** 10.3390/toxics12120864

**Published:** 2024-11-28

**Authors:** Juan Tang, Kai Wang, Dan Shen, Chunmei Li

**Affiliations:** 1 College of Animal Science and Technology, Nanjing Agricultural University, Nanjing 210095, China; tangjuanttjj@163.com (J.T.); wk@stu.njau.edu.cn (K.W.); chunmeili@njau.edu.cn (C.L.); 2 Department of Occupational Medicine and Environmental Toxicology, Nantong Key Laboratory of Environmental Toxicology, School of Public Health, Nantong University, Nantong 226019, China

**Keywords:** bisphenol A, hepatotoxicity, HO-1, Nrf2, rat

## Abstract

Bisphenol A (BPA), extensively utilized in the manufacture of epoxy resins and polycarbonate plastics, is prevalent in the environment. Its exposure has been associated with an increased risk of hepatic lesions; however, the underlying mechanisms and the spectrum of its effects remain poorly understood. This study investigates the role of the Keap1-Nrf2 signaling pathway in regulating BPA-induced hepatotoxicity in vivo using a rat model. Over a 30-day period, rats were orally administered either corn oil or BPA (0.5, 5, and 50 mg/kg). Changes in hepatic and kidney histology were assessed via transmission electron microscopy and HE staining. Oxidative stress levels in the liver tissue and serum were quantified, while the mRNA expression of *Nrf2*, *Keap1*, *GPX2*, *HO-1*, and *caspase-3* was evaluated using qRT-PCR. Additionally, the expression of Nrf2 and cleaved caspase-3 in the liver tissue was measured through immunohistochemistry and Western blotting. Results indicated that BPA exposure significantly reduced the liver and adrenal coefficients in the treated rats compared to controls. Notable histomorphological alterations were observed in the liver and kidney tissues of the BPA-treated rats. The serum levels of GOT and TNF-α were significantly elevated in the BPA group relative to the controls. Evidence of oxidative stress was supported by increased malondialdehyde levels and decreased total superoxide dismutase activity in the liver and kidney, alongside a reduction in glutathione peroxidase activity in the liver tissue. Furthermore, BPA exposure enhanced the mRNA expression levels of *Nrf2*, *Keap1*, *GPX2*, *HO-1*, and *caspase-3* in the liver tissue. Concurrently, Nrf2 and cleaved caspase-3 expression levels were elevated in the BPA-treated group compared to the controls. These findings suggest that BPA may contribute to metabolic disorders of liver function and poses a hepatotoxicity risk. Moreover, the activation of the Keap1-Nrf2 pathway may offer protective effects against hepatotoxicity, with potential implications for human liver disease.

## 1. Introduction

Bisphenol A (BPA) is a significant chemical raw material used in the production of epoxy resins and polycarbonate plastics. It is commonly utilized in the manufacturing of food packaging, including the linings of canned food containers, beverage packaging, and bottles, as well as dental materials [1]. Importantly, BPA polymers in food packaging can leach into food products under conditions such as high temperatures or acidity, leading to elevated exposure levels in humans [2]. Due to its persistence in the environment and widespread distribution [3,4], there has been increasing research focused on understanding the toxicity of BPA. Its estrogenic properties as a xenoestrogen raise concerns about its potential role in various health disorders [5,6,7]. Epidemiological studies have demonstrated positive correlations between obesity and elevated urinary BPA levels [8,9,10], as well as links to fertility impairment [11,12] and cardiovascular disease [13]. Additionally, BPA exposure has been associated with increased cancer risks [14,15]. While the primary focus of BPA research has historically centered on reproductive health, there is a growing body of evidence suggesting that BPA may also impact liver function. The liver plays a critical role in the metabolism of various xenobiotics, including BPA, and altered liver function can lead to significant metabolic disturbances. Emerging studies indicate that BPA exposure may provoke hepatotoxic effects, although this aspect remains underexplored. Understanding the mechanisms by which BPA influences liver health is crucial, as it may further elucidate the systemic implications of BPA exposure and its contribution to broader metabolic disorders. Notably, previous investigations have indicated that BPA may exhibit both reproductive toxicity and hepatotoxicity in rat models [16,17].

Nuclear factor E2-related factor 2 (Nrf2) primarily serves as the cell’s antioxidant defense [18] and is involved in responses to cellular stress [19]. Oxidants and electrophiles can modify critical cysteine residues in the Kelch ECH-associating protein 1 (Keap1), leading to a conformational change that disrupts its ability to ubiquitinate Nrf2, thereby stabilizing and activating Nrf2 [20]. The antioxidant response element (ARE) in the regulatory regions of target genes is bound by Nrf2 and tiny Maf proteins, and Keap1 binds to Nrf2 and facilitates its destruction by the ubiquitin proteasome pathway. Nrf2-bound Keap1 is deactivated, and newly formed Nrf2 proteins bypass Keap1 and move into the nucleus where they attach to the ARE and activate Nrf2 target genes such as heme oxygenase 1 (HO-1) to produce proteins. The Nrf2-Keap1 pathway controls the main antioxidant and cytoprotective responses to exogenous and endogenous stressors and offers critical protection against oxidative stress [21].

In the current work, we show that BPA has negative impacts on hepatic histomorphology in rats, as well as affecting the oxidative and antioxidant metabolism balance in their liver tissue. We know very little about how BPA exposure affects the expression of Keap1 and Nrf2 in male rats’ liver tissues. The goal of this study was to determine whether BPA exposure may lead to the development of hepatic damage, which may be linked to genes connected with the Keap1-Nrf2 signaling pathway. Collectively, these results show that the Keap1-Nrf2 pathway can be engaged in the liver and provide protection against hepatic oxidative stress and inflammation injury to the liver.

## 2. Materials and Methods

### 2.1. Animals and Treatment

From the Nanjing Qinglongshan Experimental Animal Center, we purchased twenty-four adult rats (Sprague Dawley, male, 8 wk-of-age, 241 ± 5.12 g) (Nanjing, China). The rats were kept in standard housing conditions (23 ± 2 °C, 50 ± 10% relative humidity, 12 h light–dark cycle). The Committee of Animal Research Institute, Nanjing Agricultural University, China, and the National Institute of Health Guidelines for Animal Care both examined and approved the experimental procedures (SYXK(Su)2021-0086). The animals were treated with compassion and consideration for a reduction in pain.

Bisphenol A (BPA), as a white powder (C15H16O2, >99% purity, CAS: 80-05-7), was bought from Sigma-Aldrich (St. Louis, MO, USA). The BPA was dissolved in corn oil and administered orally for 30 days. The daily dose used was 0.5, 5, and 50 mg/kg [22]. The rats were weighed daily, and their average body weight was determined as the average of each rat’s individual body weight in each group every day.

### 2.2. Histopathologic Evaluation

For the determination of the liver and kidney pathological changes, the liver and kidney tissues were fixed in 4 % formaldehyde solution for 24 h, embedded in paraffin. The paraffin was sectioned (4-μm) and stained with haematoxylin–eosin (HE) for microscopic examination. The hepatic and renal damages were evaluated by using the score system [23]. The scores program has been reported by our previous studies [22]. Briefly, the scores of liver sections are graded on a 0–4 scale for lobular inflammation, focal necrosis, and mononuclear cell infiltration, and the kidney is graded on a 0–4 scale for proximal and distal tubular necrosis, glomerular cellularity, and glomerular necrosis (where 0 represents no abnormality, and 1, 2, 3, and 4 represent mild, moderate, moderately severe, and severe abnormalities, respectively).

### 2.3. Transmission Electron Microscopy

Liver and kidney tissues were obtained from the animals and fixed in 2.5% glutaraldehyde for 24 h and then dried in a series of progressively stronger alcohols, purified using xylene, and embedded in paraffin.

For transmission electron microscopy analysis, 70 nm sections were mounted on copper grids and stained with uranyl acetate and lead citrate. The method has been reported by previous studies [24].

### 2.4. Biochemical Evaluation

An automatic biochemical analyzer was used to test the levels of the liver damage indicators glutamic–oxalacetic transaminase (GOT) and glutamic–pyruvic transaminase (GPT), which were expressed as U/L blood. A commercially available enzyme-linked immunosorbent assay (ELISA) was used to measure the levels of interleukin-1 (IL-1), IL-6, and tumor necrosis factor (TNF-α) in the serum and liver tissue. We bought the kits from R&D Systems (Shanghai, China). Using commercial reagent kits procured from the Institute of Biological Engineering of Nanjing Jiancheng (Nanjing, China), superoxide dismutase (SOD), malondialdehyde (MDA) content, glutathione (GSH), catalase (CAT), and glutathione peroxidase (GSH-Px) activities were all measured.

### 2.5. Immunohistochemistry

Using formalin-fixed liver tissue, the expression of Nrf2 and cleaved caspase-3 was examined for immunohistochemistry analysis. To quench endogenous peroxidase, the liver sections (5 μm thick) were deparaffinized, hydrated, rinsed, and incubated. The primary antibodies were the rabbit polyclonal Nrf2 and cleaved caspase-3 (diluted 1:100) (Beyotime Biotechnology, China; diluted 1:100). According to the manufacturer’s recommendations, biotinylated goat anti-rabbit IgG secondary antibody was used to detect immunoreactivity, and then avidin-biotinylated horseradish peroxidase complex was observed using diaminobenzidine tetrahydrochloride. Sections were counterstained with hematoxylin. Every test had a positive and a negative control, and the negative controls were made by omitting the primary antibodies. The stained sections were observed and photographed using NIS-Elements BR 2.30. ImageJ 1.50d was used to detect and calculate the positive cell rate.

### 2.6. Western Blotting

We used a protein extraction kit (Beyotime Institute of Biotechnology (Nantong, China), P0027) for the 50 mg live tissue, and liver proteins were extracted in accordance with the manufacturer’s recommendations. A BCA protein assay kit was used to measure the total protein content (Thermo Fisher Scientific, Rockford, IL, USA). After electrophoresis on a 12% SDS-PAGE gel, the separated proteins (50 g) were transferred to nitrocellulose membranes. Defatted milk powder was used to incubate the membranes in order to prevent non-specific binding. The primary antibodies were incubated: Nrf2 and cleaved caspase-3 (1:1000, Cell Signaling Technology, Boston, MA, USA). After the membranes were cleaned, they were incubated with the secondary antibody: HRP-labeled goat anti-rabbit IgG. A luminescent image analyzer (Scion Image v. 4.0.2, Scion Corporation, Frederick, MD, USA) was used to visualize and analyze the Western blot bands. β-actin served as the reference. Image J was used to accomplish the densitometry.

### 2.7. RNA Preparation and mRNA Quantification

Total liver RNA was extracted by using TRIzol reagent (Invitrogen, Waltham, MA, USA) in accordance with the manufacturer’s instructions. The RNeasy Mini Kit with the RNase-Free DNase Set (Qiagen, Valencia, CA, USA) was used to purify the RNA samples, and cDNA was generated with the Omniscript^®^ Reverse Transcription kit (Takara, Kusatsu, Japan) with Oligo-dT primers (Takara). We quantitated gene expression in triplicate by real-time PCR using a QuantiTectTMSYBR Green^®^ PCR Kit (Roche, Basel, Switzerland). The results of the gene expression were adjusted to account for β-actin expression. The primer sequences are displayed in Table 1. The primers’ design was conducted using Primes Premier 5.0 and synthesized by Shanghai Generay Biotech Co., Ltd. (Shanghai, China) Gene expression changes were quantified using the Ct (2^−ΔΔCt^) method [25].

### 2.8. Statistical Analysis

The results are shown as mean ± SD. GraphPad Prism 10 software was used for the data analysis, which included the unpaired Student’s *t*-test, repeated-measures one-way analysis of variance (ANOVA), and Tukey’s multiple comparison tests (GraphPad Software, San Diego, CA, USA). We considered a *p*-value of <0.05 statistically significant.

## 3. Results

### 3.1. Body and Organ Weights

The results showed that the liver weight decreased significantly in the 50 mg/kg BPA treatment groups compared to the control group, and the liver and adrenal coefficient of rats in the 5 mg/kg and 50 mg/kg BPA treatment groups decreased significantly compared to the corresponding control group (Table 2). Nevertheless, compared to the control group, there was no discernible variation in the weight of other organs or their relative indices (Table 2).

### 3.2. Histopathological Evaluation

**Figure 1:**
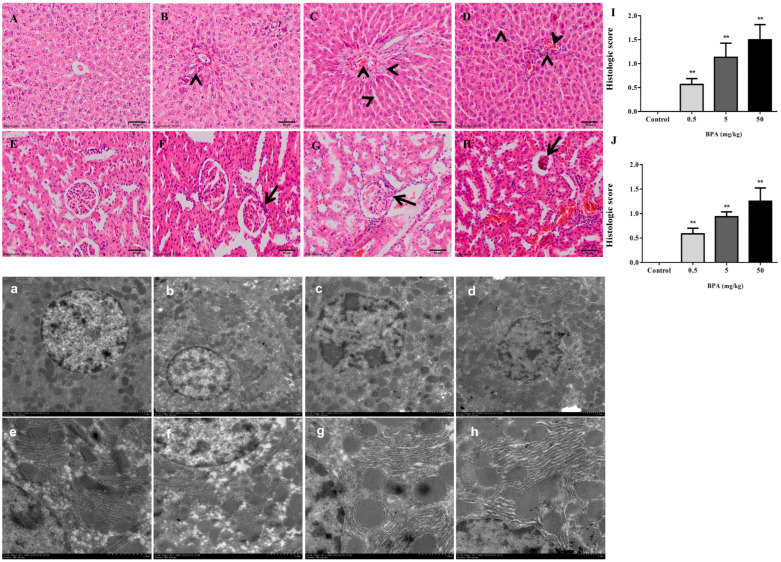
Histopathological changes in the liver and kidney tissues of male rats following oral BPA administration through hematoxylin and eosin staining at a 200× magnification. (**A**) A normal liver section. (**B**–**D**) BPA-treated groups (0.5, 5, and 50 mg/kg). (**E**) A normal kidney section. (**F**–**H**) BPA-treated groups (0.5, 5, and 50 mg/kg). (**I**,**J**) The hepatic and renal damages histologic score evaluation compared to the control; ** *p* < 0.01. Electron micrographs of liver tissues from control and BPA-treated rats. (**a**,**e**) A normal liver section. (**b**–**d**,**f**–**h**) BPA-treated groups (0.5, 5, and 50 mg/kg).

The histological evaluation of the liver and kidney sections are shown in Figure 1. The normal rat liver tissue displayed hepatic lobules with a core vein surrounded by radiating hepatocytes (Figure 1A). On the other hand, following BPA exposure, all BPA treatment groups showed some signs of liver damage. Rats given the BPA treatment had localized necrosis, mononuclear cell infiltration, and some hepatocytes that underwent apoptosis. Compared to the control group, the BPA-exposed rats’ liver tissue exhibited varied cellular vacuolization, monocyte infiltration, and hydropic degeneration (Figure 1B–D). Rats in the 0.5 mg/kg BPA-treated groups showed modest periportal enlargement and hepatocyte death in their livers (Figure 1B). Liver damage peaked following exposure to 5 mg/kg and 50 mg/kg BPA and was characterized by higher levels of structural disorder, apoptosis in certain hepatocytes, and cell infiltration by monocytes (Figure 1C,D).

The glomerulus, which is encompassed by the Bowman capsule and has convoluted tubules, was generally intact in the control rats’ renal tissue (Figure 1E). In contrast, the BPA-treated rats’ kidney sections showed tubular necrosis and glomerular toxicity (Figure 1F–H). In comparison, only minor damage was seen in the rat renal tissue with proximal and distal tubular necrosis after the 0.5 mg/kg BPA exposure (Figure 1H). However, rats exposed to 5 mg/kg and 50 mg/kg of BPA had more distal and proximal tubular necrosis and glomerular damage in their kidneys (Figure 1F,G). Moreover, there was a significant increase in the histologic score of liver and kidney damage in all the BPA-treated groups as compared to the control group (Figure 1I,J). We examined the ultrastructural alterations in the rat liver tissue to study the effects of exposure to BPA on the structure of liver cells. The ultrastructure of the rat liver tissue in shown in Figure 1a–h. The control rats showed circular hepatocyte nuclei, and there were many intact mitochondria and an endoplasmic reticulum around the nucleus, with no structural changes (Figure 1a). At the same time, the endoplasmic reticulum structure was normal and neatly arranged (Figure 1e). In the BPA-treated group, compared to the control group, the nucleus of the hepatocytes showed different degrees of pyknosis, increased mitochondria (Figure 1b–d), a disordered endoplasmic reticulum, and swelling (Figure 1f–h).

### 3.3. Assessment of Liver Function and IL-1β, IL-6, and TNF-α Levels in Serum and Liver

**Figure 2:**
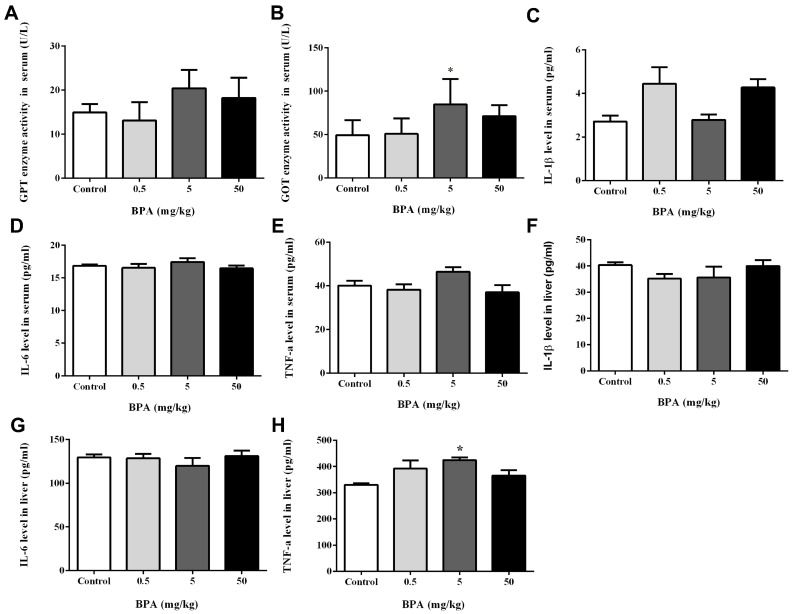
Effect of BPA treatment on GPT (**A**), GOT (**B**) enzyme activities, IL-1β (**C**), IL-6 (**D**), and TNF-α (**E**) levels in serum. Level of IL-1β (**F**), IL-6 (**G**), and TNF-α (**H**) in liver tissue. Data shown are mean ± SD of eight animals in each group compared to control; * *p* < 0.05.

To research how BPA affects liver function, we measured the GPT and GOT levels in the serum. Results revealed that the GOT level was significantly increased in the 5 mg/kg BPA treatment group compared to the control group (Figure 2B), which showed that the BPA affected hepatic metabolism. Nonetheless, we noticed a trend toward elevated GPT levels in rats exposed to 0, 5, and 50 mg/kg of BPA, even if this was not statistically significant (Figure 2A).

The concentration of the inflammatory mediators IL-1β, IL-6, and TNF-α in the serum and liver were determined as shown in Figure 2C–H. The TNF-α level showed a significant increase in the 5 mg/kg BPA-treated group compared to the control group in the liver tissue (Figure 2H).

### 3.4. Evaluation of Serum, Liver, and Kidney Enzyme Levels to Analyze Oxidative Stress

We investigated the SOD, CAT, and GSH-PX activities and the MDA concentration in the serum, livers, and kidneys to determine whether or not BPA may cause oxidative stress in vivo. Results showed that CAT activity significantly increased in the 5 and 50 mg/kg BPA treatment group compared to the control group in the serum (Table 3).

The SOD activity showed a significant decrease in the 5 mg/kg BPA-treated group compared to the control group, and the MDA content significantly increased compared to the control group in the 50 mg/kg BPA-treated group in the liver (Table 3). In addition, compared to the control group, GSH-PX activity showed a significant decrease in the 0.5 mg/kg BPA-treated group (Table 3).

As shown in Table 3, the SOD activity in all the BPA-treated groups showed a significant decrease in the kidney compared to the control group. However, compared to the control group, the 5 mg/kg BPA-treated group’s MDA content dramatically increased.

### 3.5. Immunohistochemistry of Nrf2 and Cleaved Caspase-3 in Liver

**Figure 3:**
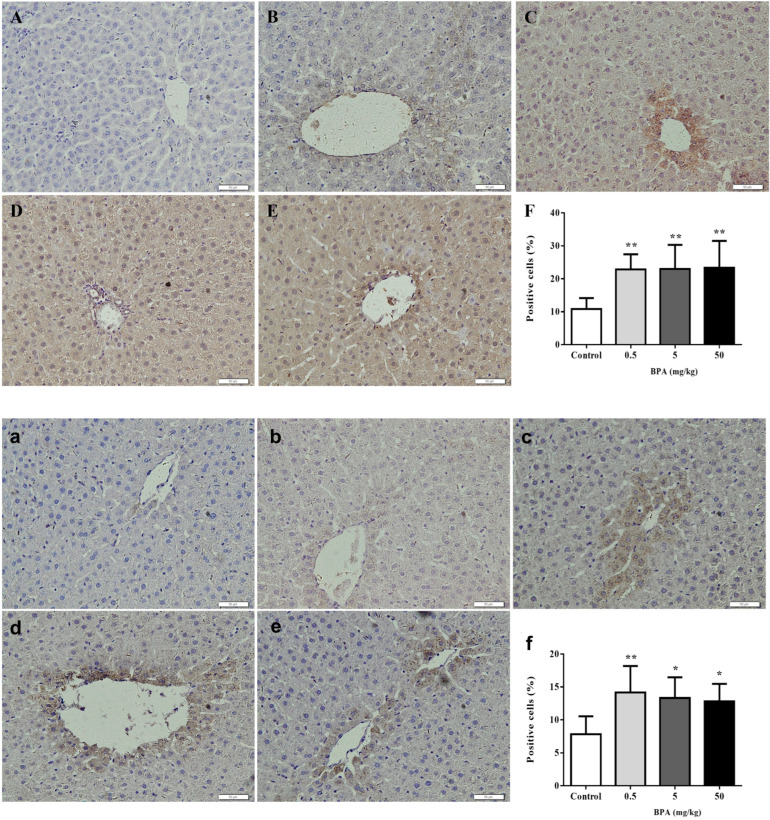
Immunohistochemical analysis of Nrf2 and cleaved caspase-3 protein in liver. Nrf2: (**A**) Negative control. (**B**) Normal liver section. (**C**–**E**) BPA-treated groups (0.5, 5, and 50 mg/kg). (**F**) Positive cells (%). Cleaved caspase-3: (**a**) Negative control. (**b**) Normal liver section. (**c**–**e**) BPA-treated groups (0.5, 5, and 50 mg/kg). (**f**) Positive cells (%). Data shown are mean ± SD of eight animals in each group compared to control; * *p* < 0.05, ** *p*< 0.01.

We examined a potential role of BPA-related alterations in the expression of Nrf2 and cleaved caspase-3, as well as a potential link between these changes and the observed liver impact. Figure 3 shows the immunohistochemical staining results for the Nrf2 and cleaved caspase-3 in different locations of the liver.

The Nrf2 protein expression was predominantly localized in the nuclei and cytoplasm of most liver tissue cells. All the BPA treatment groups significantly increased in their expression of the Nrf2 protein when compared to the control group (Figure 3A–E), and statistical analysis revealed that the percentage of Nrf2-positive cells in the liver tissue was significantly higher in the 0.5, 5, and 50 mg/kg BPA treatment groups (Figure 3F).

Also, it was discovered that the cleaved caspase-3 protein was more abundant in the liver tissue’s cytoplasm and increased in the 0.5, 5, and 50 mg/kg BPA treatment groups (Figure 3a–e). In comparison to the control group, the percentage of cleaved caspase-3-positive cells considerably increased in the 0.5, 5, and 50 mg/kg BPA treatment groups (Figure 3f).

### 3.6. Nrf2 Involved in BPA-Induced Liver Oxidative Damage in Rats

**Figure 4:**
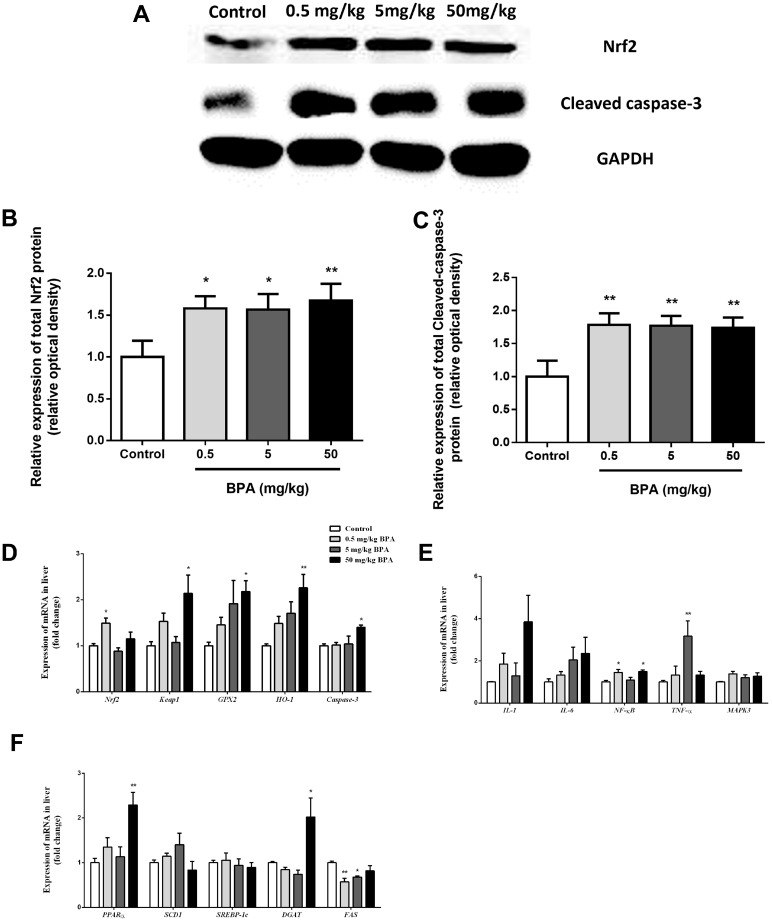
Protein expression of Nrf2 and cleaved caspase-3 in liver samples of rats. Western blot analysis of Nrf2 and cleaved caspase-3 protein in liver (**A**–**C**). RT-PCR analyses of Nrf2, Keap1, GPX2, HO-1, and caspase-3 mRNA of liver (**D**). RT-PCR analyses of IL-1, IL-6, NF-κB, TNF-α, and MAPK3 mRNA of liver (**E**). RT-PCR analyses of PPARα, SCD1, SREBP1c, DGAT, and FAS (**F**). Data shown are mean ± SEM of eight animals in each group compared to control; * *p* < 0.05, and ** *p* < 0.01.

To further expound the mechanism of the effect on BPA-induced hepatotoxicity by Nrf2, we then investigated the intervention of Nrf2 on BPA-induced liver oxidative damage in the rats. Meanwhile, the levels of cleaved caspase-3 protein expression were measured to evaluate whether the BPA triggered the cleaved caspase-3. Liver proteins were removed for Western blotting examination. Results indicated that the BPA treatment significantly increased the production of Nrf2 in the liver tissue (Figure 4A,B). When compared to the control group, cleaved caspase-3 levels in the liver tissue significantly increased in the BPA treatment groups (Figure 4A,C). The Nrf2 mRNA expression level in the 0.5 mg/kg BPA treatment group showed a significant increase when compared to the control group (Figure 4D). At the same time, the Keap1, GPX2, HO-1 and caspase-3 mRNA expression were significantly increased in the 50 mg/kg BPA treatment group compared to the control group (Figure 4D). The expression of IL-1, IL-6, NF-κB, TNF-α, and MAPK3 mRNA was quantitatively determined using real-time PCR in the liver affected by the BPA treatment (Figure 4E). Compared to the control group, the RT-PCR results revealed that the NF-κB mRNA expression in the liver tissue increased significantly in the 0.5 and 50 mg/kg BPA treatment groups, and the expression of TNF-α mRNA in the 5 mg/kg BPA treatment groups significantly increased (Figure 4E). Although there was a trend toward increased IL-1 and IL-6 mRNA expression in the BPA treatment group, the difference from the control group was not statistically significant (Figure 4E). We examined the effects of BPA involved in the lipid metabolism in the liver tissue (Figure 4F). After the 50 mg/kg BPA exposure, the hepatic peroxisome proliferator-activated receptor alpha (PPARα) and diacylglycerol O-acyltransferase (DGAT) mRNA expression considerably increased in comparison to the control group. Moreover, we observed a substantial reduction in the FAS mRNA expression in the groups treated with 0.5 and 5 mg/kg of BPA in comparison to the control group (Figure 4F).

## 4. Discussion

This investigation elucidates the detrimental effects of bisphenol A (BPA) on oxidative stress and the expression of key regulatory genes—Nrf2, Keap1, GPX2, and HO-1—in rat liver tissue. Our findings indicate that BPA induces significant hepatic oxidative injury, while the enhanced biotransformation of Nrf2 may confer a protective effect against BPA-induced hepatotoxicity. Furthermore, our data suggest that the Nrf2 signaling pathway is instrumental in modulating hepatotoxic responses in BPA-exposed rats.

BPA is pervasive in various consumer products, including beverage cans, dental materials, and thermal paper [26]. The literature has documented the adverse effects of BPA on reproductive health, neurodevelopment, and glucose homeostasis and its potential contribution to obesity and metabolic syndrome [27,28,29,30]. Notably, our research found no significant impact of BPA on the developmental capacities of the rat population; however, the BPA-treated rats exhibited marked reductions in their liver and adrenal coefficients. Given BPA’s established role as a risk factor for numerous diseases [31,32] and the chronic low-dose exposure experienced by the general population [5], it is imperative to explore its implications for liver toxicity.

Previous studies have corroborated BPA’s potential to induce hepatic injury [33,34,35]. In our current study, we assessed the impact of BPA on both liver and kidney tissues, revealing that BPA exposure significantly elevated serum GOT levels and was associated with substantial hyperemia, cellular degeneration, necrosis, and renal tubular injury, as well as impaired glomerular filtration, in the hepatic and renal cells of rats. Ultrastructural analyses further demonstrated varying degrees of pyknosis in hepatocyte nuclei, increased mitochondrial proliferation, a disordered endoplasmic reticulum, and cytoplasmic swelling in the BPA-treated livers. Although our observations confirmed hepatic injury following the BPA exposure, the precise mechanisms underlying this phenomenon remain elusive. The role of oxidative stress in BPA-induced hepatotoxicity has been emphasized in prior research [35,36], suggesting that BPA may disrupt liver function through oxidative and pro-inflammatory pathways.

Numerous studies have established a link between BPA exposure and oxidative stress, leading to liver damage, inflammation, and apoptotic cascades [37,38,39]. An in vivo investigation has demonstrated that BPA can provoke liver damage via the generation of reactive oxygen species (ROS), which compromise hepatic integrity [40]. In our study, exposure to BPA resulted in significant reductions in the activities of critical antioxidant enzymes, specifically SOD and GSH-PX. In contrast, we observed significant increases in the MDA content and CAT activity. Additionally, pro-inflammatory cytokines and chemokines, such as IL-1β, IL-6, and TNF-α, have been produced by hepatic macrophages in various murine models of liver injury [41,42]. Our results corroborate these findings, demonstrating a significant increase in TNF-α levels in liver tissues following BPA exposure.

The mechanisms by which BPA induces oxidative stress and inflammation remain to be fully elucidated. Research by Deshmukh et al. suggests that the Nrf2-Keap1 signaling pathway is a critical regulator of oxidative stress responses [43]. BPA exposure induces oxidative stress, as evidenced by elevated TNF-α levels, an increased MDA content, and decreased antioxidant enzyme activity in liver tissues. While Nrf2 activation represents a cellular defense mechanism against such stress, the balance between oxidative damage and protective responses may vary depending on the intensity and duration of the exposure [44,45]. Nrf2 is hypothesized to modulate the cellular cycle of hepatocytes [46] through both canonical and non-canonical activation pathways. The canonical pathway involves the dissociation of Nrf2 from its inhibitor, Keap1, leading to its translocation into the nucleus, where it orchestrates the cellular antioxidant response, encompassing antioxidant defenses, detoxification, and redox homeostasis [47]. The Keap1-Nrf2 signaling axis regulates cellular and organismal susceptibility to oxidative insults by controlling the basal expression of a diverse array of protective genes [48,49,50]. PPARα is highlighted as a key regulator of lipid metabolism and oxidative stress, while DGAT is involved in triglyceride synthesis, both of which are relevant to BPA-induced hepatic effects. In summary, our findings suggest that the hepatotoxicity induced by BPA exposure may be mediated through the activation of Nrf2 signaling pathway-associated genes, highlighting the need for further investigation into the molecular mechanisms underlying BPA-induced liver damage.

This study provides valuable insights into the hepatotoxic effects of BPA and the potential protective role of the Keap1-Nrf2 pathway; however, several limitations should be acknowledged. While key oxidative stress markers and histological changes were assessed, this study lacks a more detailed exploration of other molecular pathways and biomarkers that may also contribute to BPA-induced hepatotoxicity.

## 5. Conclusions

In general, the current work explores the mechanisms of BPA exposure and the development of rat liver oxidative damage. These alterations in hepatic oxidative damage might result from a destroyed oxidation and antioxidant balance in hepatocytes through increasing related gene expression.

## Figures and Tables

**Table 1:** Primers used for quantitative real-time PCR.

Gene Symbol	Accession No.	Primer Sequence (5′ to 3′)	Product Size (bp)	40 PCR Cycles
IL-1	NM_031512.2	F: GCCAACAAGTGGTATTCTCCA R: TGCCGTCTTTCATCACACAG	120	95 °C for 15 s 60 °C for 30 s 72 °C for 30 s
IL-6	NM_012589.2	F: AGTTGCCTTCTTGGGACTGA R: ACTGGTCTGTTGTGGGTGGT	102	95 °C for 15 s 60 °C for 30 s 72 °C for 30 s
MAPK3	NM_017347.2	F: CTACACGCAGCTGCAGTACATC R: GTGCGCTGACAGTAGGTTTGA	153	95 °C for 15 s 60 °C for 30 s 72 °C for 30 s
NF-kB	NM_001276711.1	F: CGACGTATTGCTGTGCCTTC R: TTGAGATCTGCCCAGGTGGTA	198	95 °C for 15 s 60 °C for 30 s 72 °C for 30 s
TNF-α	NM_012675.3	F: TTCCGTCCCTCTCATACACTG R: AGACACCGCCTGGAGTTCT	149	95 °C for 15 s 60 °C for 30 s 72 °C for 30 s
Keap1	NM_057152.2	F: CATCGGCATCGCCAACTTC R: GCTGGCAGTGTGACAGGTTGA	278	95 °C for 15 s 60 °C for 30 s 72 °C for 30 s
GPx2	NM_183403.2	F: CCGTGCTGATTGAGAATGTG R: AGGGAAGCCGAGAACCACTA	113	95 °C for 15 s 60 °C for 30 s 72 °C for 30 s
Caspase-3	NM_012922	F: AAGCCGAAACTCTTCATC R: TGAGCATTGACACAATACAC	349	95 °C for 15 s 60 °C for 30 s 72 °C for 30 s
PPARα	NM_001145367.1	F: CTCGTGCAGGTCATCAAGAA R: CAGCCCTCTTCATCTCCAAG	158	95 °C for 15 s 60 °C for 30 s 72 °C for 30 s
DGAT	NM_053437.1	F: TCTTCCTACCGGGATGTCAATC R: TCCCTGCAGACACAGCTTG	204	95 °C for 15 s 60 °C for 30 s 72 °C for 30 s
SREBP1c	NM_001271207.1	F: GCCATGGATTGCACATTG R: TGTGTCTCCTGTCTCACCCC	187	95 °C for 15 s 60 °C for 30 s 72 °C for 30 s
SCD1	NM_009127.4	F: CCTTAACCCTGAGATCCCGTAGA R: AGCCCATAAAAGATTTCTGCAAA	237	95 °C for 15 s 60 °C for 30 s 72 °C for 30 s
FAS	NM_139194.2	F: GGACATGGTCACAGACGATGAC R: GGAGGCGTCGAACTTGGA	279	95 °C for 15 s 60 °C for 30 s 72 °C for 30 s
Nrf2	NM_010902	F: ACAGATGGCGTCACTTCG R: TGAGGACCCACTGGAGGA	109	95 °C for 15 s 60 °C for 30 s 72 °C for 30 s
HO-1	NM_010442	F: ACAGATGGCGTCACTTCG R: TGAGGACCCACTGGAGGA	128	95 °C for 15 s 60 °C for 30 s 72 °C for 30 s
β-actin	NM_031144.3	F:AGCCATGTACGTAGCCATCC R:CTCTCAGCTGTGGTGGTGAA	227	95 °C for 15 s 60 °C for 30 s 72 °C for 30 s

Note: interleukin-1 (IL-1), interleukin-6 (IL-6), mitogen-activated protein kinase 3 (MAPK3), nuclear factor-kappa B (NF-κB), tumor necrosis factor-alpha (TNF-α), Kelch-like ECH-associated protein 1 (Keap1), glutathione peroxidase 2 (GPx2), caspase-3 (caspase-3), peroxisome proliferator-activated receptor alpha (PPARα), diacylglycerol O-acyltransferase (DGAT), sterol regulatory element-binding protein 1c (SREBP1c), stearoyl-CoA desaturase 1 (SCD1), fatty acid synthase (FAS), nuclear factor erythroid 2-related factor 2 (Nrf2), heme oxygenase-1 (HO-1), beta-actin (β-actin).

**Table 2:** Body weights and organ weights of male rats treated with BPA for 30 days.

	Control	GLP (mg/kg Body Weight)		
	0	0.5	5	50
Number of animals	6	6	6	6
Initial body weight (g)	235.30 ± 5.65	232.70 ± 4.48	234.60 ± 5.04	236.90 ± 4.45
Body weight (g)	362.20 ± 16.82	360.20 ± 3.92	367.30 ± 11.10	357.10 ± 4.28
Body weight gain (g)	126.80 ± 11.50	127.50 ± 3.70	132.70 ± 9.72	120.30 ± 4.87
Average daily gain(g)	4.23 ± 0.384	4.25 ± 0.12	4.42 ± 0.32	4.01 ± 0.16
Liver weight (g)	13.34 ± 0.91	12.52 ± 0.20	11.90 ± 0.40	10.94 ± 0.30 *
Liver index (%)	3.67 ± 0.11	3.48 ± 0.04	3.24 ± 0.06 **	3.07 ± 0.09 **
Kidney weight (g)	2.34 ± 0.14	2.42 ± 0.10	2.41 ± 0.07	2.47 ± 0.05
Kidney index (%)	0.65 ± 0.03	0.67 ± 0.03	0.66 ± 0.02	0.69 ± 0.02
Spleen weight (g)	0.73 ± 0.06	0.68 ± 0.04	0.71 ± 0.07	0.74 ± 0.03
Spleen index (%)	0.20 ± 0.01	0.19 ± 0.01	0.19 ± 0.01	0.21 ± 0.01
Heart weight (g)	1.38 ± 0.07	1.60 ± 0.09	1.50 ± 0.10	1.45 ± 0.08
Heart index (%)	0.39 ± 0.03	0.44 ± 0.03	0.41 ± 0.03	0.41 ± 0.02
Lung weight (g)	1.69 ± 0.25	1.54 ± 0.06	1.47 ± 0.08	1.56 ± 0.09
Lung index (%)	0.41 ± 0.03	0.43 ± 0.02	0.40 ± 0.02	0.44 ± 0.03
Adrenal weight (g)	0.08 ± 0.02	0.069 ± 0.017	0.047 ± 0.006	0.049 ± 0.005
Adrenal index (%)	0.03 ± 0.01	0.014 ± 0.001 *	0.013 ± 0.002 *	0.014 ± 0.001 *

Note: The values shown are the mean ± SEM of eight animals per group, compared to the control; * *p* < 0.05 and ** *p* < 0.01.

**Table 3:** Effects of BPA on oxidative stress parameters in serum, liver, and kidney of rats.

	Control	BPA (mg/kg Body Weight)		
	0	0.5	5	50
Serum				
SOD (U/mL)	655.50 ± 28.61	608.50 ± 9.19	681.00 ± 23.10	702.70 ± 23.05
MDA (nmol/mL)	7.57 ± 0.95	8.25 ± 0.96	10.60 ± 1.08	9.93 ± 1.28
CAT (U/mL)	36.10 ± 4.89	46.08 ± 4.40	71.39 ± 7.90 **	64.90 ± 6.58 **
GSH-PX (U/L)	85.26 ± 10.03	98.53 ± 15.54	103.80 ± 19.94	66.74 ± 22.16
Liver				
SOD (U/mgprotein)	287.30 ± 17.70	222.90 ± 23.79	210.50 ± 6.50 *	235.1 ± 24.36
MDA (nmol/mgprotein)	1.30 ± 0.15	1.69 ± 0.17	1.97 ± 0.24	2.20 ± 0.38 *
CAT (U/mgprotein)	42.03 ± 5.69	42.46 ± 11.21	26.83 ± 4.81	30.74 ± 7.18
GSH-PX (U/mgprotein)	610.40 ± 72.82	239.90 ± 45.61 *	397.90 ± 87.11	427.80 ± 117.30
Kidney				
SOD (U/mgprotein)	147.00 ± 7.02	89.52 ± 5.74 **	86.15 ± 7.50 **	78.08 ± 2.06 **
MDA (nmol/mgprotein)	1.59 ± 0.26	1.85 ± 0.12	3.39 ± 0.49 **	2.44 ± 0.31
CAT (U/mgprotein)	45.55 ± 3.64	40.03 ± 1.22	39.56 ± 2.62	42.73 ± 1.91
GSH-PX (U/mgprotein)	962.90 ± 185.40	728.90 ± 116.00	805.10 ± 121.30	782.20 ± 207.20

Note: superoxide dismutase (SOD), malondialdehyde (MDA) content, catalase (CAT), and glutathione peroxidase (GSH-Px) The values shown are the mean ± SEM of eight animals per group, compared to the controls; * *p* < 0.05 and ** *p* < 0.01.

## Data Availability

All data are presented in this manuscript.
